# Tildrakizumab in moderate‐to‐severe plaque psoriasis: A multicenter, retrospective, real‐life study

**DOI:** 10.1111/dth.15488

**Published:** 2022-04-11

**Authors:** Giacomo Caldarola, Marco Galluzzo, Nicoletta Bernardini, Laura Calabrese, Marta Grimaldi, Gaia Moretta, Gianluca Pagnanelli, Ruslana Gaeta Shumak, Marina Talamonti, Lorenzo Tofani, Sabatino Pallotta, Ketty Peris, Concetta Potenza, Clara De Simone, Elena Campione

**Affiliations:** ^1^ Dermatologia, Dipartimento di Medicina e Chirurgia Traslazionale Università Cattolica del Sacro Cuore Rome; ^2^ UOC di Dermatologia, Dipartimento di Scienze Mediche e Chirurgiche Fondazione Policlinico Universitario A. Gemelli – IRCCS Rome; ^3^ Department of Systems Medicine University of Rome “Tor Vergata” Rome Italy; ^4^ Dermatology Unit Fondazione Policlinico “Tor Vergata” Rome Italy; ^5^ Department of Medical‐Surgical Sciences and Biotechnologies, Dermatology Unit “Daniele Innocenzi” Sapienza University of Rome Rome Italy; ^6^ Istituto Dermopatico dell'Immacolata – IRCCS Rome Italy

**Keywords:** efficacy, interleukin‐23, psoriasis, real‐life, safety, tildrakizumab

## Abstract

New biologic agents targeting interleukin (IL)23/T‐helper17 axis, such as tildrakizumab, have been developed for the treatment of plaque psoriasis. To analyze the efficacy and safety of tildrakizumab in a real life setting of patients affected by moderate‐to‐severe psoriasis over a 28‐week treatment period. A multicentric retrospective study was conducted in patients who initiated tildrakizumab between February 2020 and March 2021. Psoriasis Area and Severity Index—PASI was measured at baseline and after 4, 16 and 28 weeks. The percentage change in PASI value from baseline to the considered time‐points, proportion of patients with absolute PASI <3 at week 28 and the percentages of achieving a PASI75 or PASI90 response were assessed. Data about potential safety issues and adverse events (AEs) were collected. Statistical analysis were performed for establish clinical efficacy and for variables predicting clinical response. Fifty nine patients with psoriasis were included. Overall mean PASI percentage reduction was of 88% from baseline to week 28 and 47 out of 59 patients (79.7%) at week 28 had an absolute PASI <3. PASI75 and PASI90 responses at week 28 were achieved by 48 (81.40%) patients and 38 (64.4.0%) patients, respectively. No substantial associations between gender, body mass index ‐ BMI, PASI at baseline and prior exposition to biological therapies and the efficacy endpoints were retrieved. No serious safety issues or discontinuations related to adverse events were reported. In our real‐life study, tildrakizumab showed high efficacy and a favorable safety profile, regardless of patient‐ and disease‐related factors.

## 1 INTRODUCTION

Following the increasing understanding of the pathogenetic mechanisms, the main therapeutic target in psoriasis has become the inhibition of the interleukin (IL)23/T‐helper17 axis, with the development of several new drugs directed against the action of IL23 and IL17.^1^, ^2^ These new drugs include tildrakizumab, a humanized Immunoglobulin G (IgG)1/k monoclonal antibody that specifically binds to the p19 protein subunit of the IL23. Several phase III trials investigated the efficacy and safety of tildrakizumab, showing superior efficacy to both placebo and etanercept together with a favorable safety profile.^3^ In reSURFACE 1, at week 28, 80.4% and 51.6% of patients treated with tildrakizumab 100 mg achieved a reduction of 75 and 90% of Psoriasis Area and Severity Index – PASI (PASI75 and PASI90 response), respectively. In reSURFACE 2, actively comparing tildrakizumab with etanercept, 73.5% and 55.5% of patients treated with tildrakizumab 100 mg achieved a PASI75 and PASI90 response at week 28 versus 53.6% and 29.4% of patients treated with etanercept. In both reSURFACE trials, clinical response appeared to be maintained over time, with 58% of patients maintaining a PASI90 response at week 64 in reSURFACE 1 and 78.4% of patients with a PASI90 response at week 52 in reSURFACE 2.

However, efficacy and safety data recorded in randomized controlled trials (RCTs) may differ from data deriving from clinical daily practice, in which demographical variability and heterogenous clinical characteristics of treated patients can influence clinical response. Thus, data retrieved from real‐life experiences, integrated with those obtained from RCTs, are essential to optimize patient's therapeutic management. To date, few real‐life studies investigating the efficacy and safety of tildrakizumab are available.^4^, ^5^, ^6^ The aim of this multicenter, retrospective study was to analyze the efficacy and safety of tildrakizumab in a population of patient from Lazio region in Italy affected by moderate‐to‐severe psoriasis, over a 28‐week treatment period.

## 2 MATERIALS AND METHODS

A retrospective analysis was performed in a cohort of patients affected by chronic plaque psoriasis, with or without psoriatic arthritis, who initiated treatment with tildrakizumab 100 mg between February 2020 and March 2021. The study population consisted of patients attending the outpatient clinics of the four participating centers (Fondazione Policlinico Universitario A. Gemelli IRCCS; Università di Roma La Sapienza – Polo Pontino; Istituto Dermopatico dell'Immacolata – IDI; Policlinico Tor Vergata) in Lazio, Italy. All enrolled patients were >18 years old. Patients with generalized or palmoplantar pustular psoriasis, erythrodermic psoriasis or who had started treatment within a clinical trial were excluded, as well as patients concurrently treated with other systemic therapies. Tildrakizumab was administered following the approved dosage of European Medicines Agency (EMA) label (100 mg at week 0, 100 mg at week 4 and then 100 mg every 12 weeks), and no dose or frequency variations were permitted. For each patient included, demographic and clinical data (age, gender, weight, height, body mass index [BMI], age of onset and duration of psoriasis, familiarity, comorbidity), previous therapeutic history (phototherapy, cyclosporine, acitretin, methotrexate and biological therapies) were collected at the time of initiation of biological therapy. Moreover, data regarding the last conventional or biological treatment before starting tildrakizumab were collected, as well as the duration of the treatment with tildrakizumab and reasons of potential drug withdrawal. PASI score^7^ measured at baseline and after 4, 16 and 28 weeks, respectively, was used to assess disease severity. Data regarding adverse events (AEs), including mild and serious adverse events, or potential safety issues were collected from each patient's medical chart. The entire study was conducted according to the principles of the Helsinki Declaration.

### 2.1 Statistical analysis

Descriptive data were reported using absolute and relative (%) frequencies for categorical variables, and mean values and standard deviations when appropriate. Efficacy analysis was performed both by assessing the percentage change in PASI value from baseline to different time‐points (4, 16 and 28 weeks after the start of tildrakizumab) and by calculating the percentages of achieving a PASI75 or PASI90 response (i.e., whether patients reached an improvement of 75% or 90% from baseline at weeks 4, 16 or 28) in the study population. Student's *t*‐test and Mann–Whitney test were used to establish clinical significance of PASI variations and if some variables (BMI, sex, previous biological treatment, PASI at baseline) was associated with the clinical response. The role of BMI and PASI at baseline was evaluated both considering a cut‐off value of 30 and 15 respectively, both considering these variables as continue ones. Statistical significance was set at *p*‐value <0.05. Analyses were performed by using STATA 13.0 Software (StataCorp, Texas).

## 3 RESULTS

Fifty‐nine patients with moderate‐to‐severe psoriasis that started biological therapy with tildrakizumab were included in the study. The mean age was 53.54 ± 12.99 years and 34 (57.62%) were males. Mean BMI value was 27.49 ± 5.35 kg/m^2^, and 7 patients (11.86%) presented a concomitant psoriatic arthritis. Mean age of onset of psoriasis was 31.27 ± 15.65 years and 35 (59.32%) and 24 (40.68%) of patients were bio‐naïve and bio‐experienced patients, respectively. The average duration of treatment with tildrakizumab was 12.06 ± 3.60 months and mean PASI score at baseline was 14.19 ± 8.11. Further demographic and clinical data are reported in Table 1. All included patients were followed until week 28.

**TABLE 1:** Demographic and clinical characteristics of the study population

Characteristics (total *n* = 59)	*N* (%); Mean ± SD
Gender	
Male	34 (57.62)
Female	25 (42.38)
Age (years)	53.54 ± 12.99
BMI (kg/m^2^)	27.49 ± 5.35
Arthropathy	
No	52 (81.14)
Yes	7 (11.86)
Familiarity	
No	38 (64.41)
Yes	21 (35.59)
Age of onset (years old)	31.27 ± 15.65
Treatment duration (mo) with tildrakizumab	12.96 ± 3.60
Previous treatment	
Phototherapy	11 (18.64)
CyA	24 (40.67)
Methotrexate	34 (57.62)
Acitretin	8 (13.56)
Apremilast	13 (22.03)
Infliximab	2 (3.38)
Etanercept	7 (11.86)
Adalimumab	11 (18.64)
Golimumab	2 (3.38)
Certolizumab	1 (1.69)
Ustekinumab	6 (10.17)
Secukinumab	9 (15.25)
Ixekizumab	4 (6.78)
Guselkumab	1 (1.69)
Brodalumab	2 (3.38)
Last biological treatment	
Naïve	35 (59.32)
Anti‐TNFα	8 (13.56)
Anti‐IL17	10 (16.94)
Anti‐IL23 or Anti‐IL12/23	4 (6.78)
Comorbidities	
Hypertension	20 (33.90)
Diabetes	12 (20.33)
Dyslipidemia	30 (50.84)
Others	11 (18.64)

Abbreviations: BMI, body mass index; CyA, cyclosporine A; SD, standard deviation.

**FIGURE 1:**
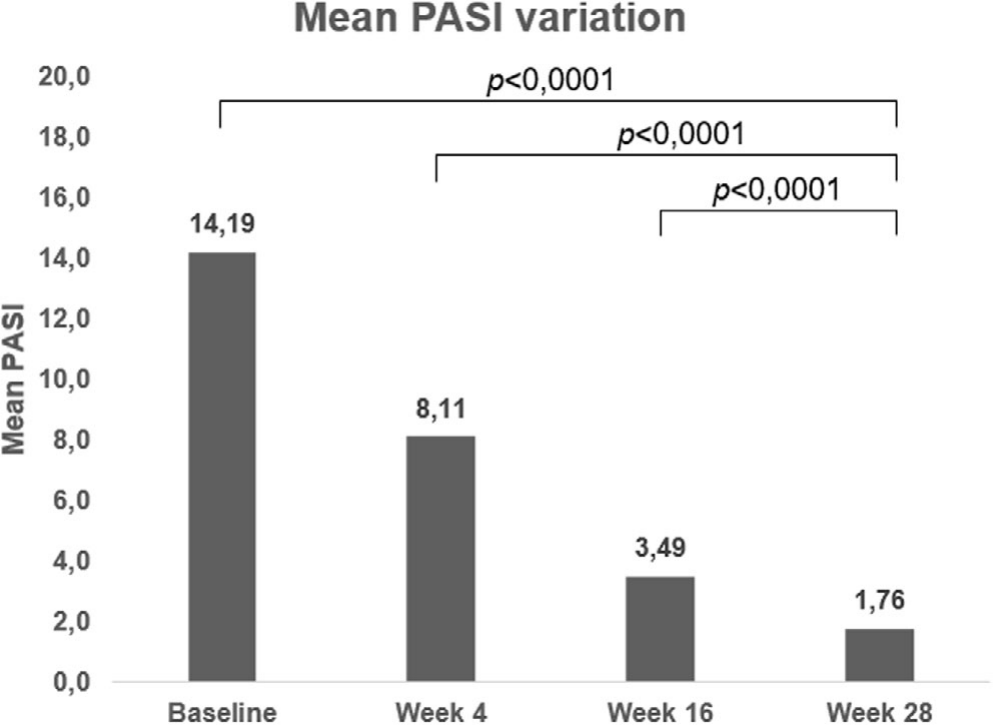
Mean PASI variation at baseline, week 4, 16 and 28

**FIGURE 2:**
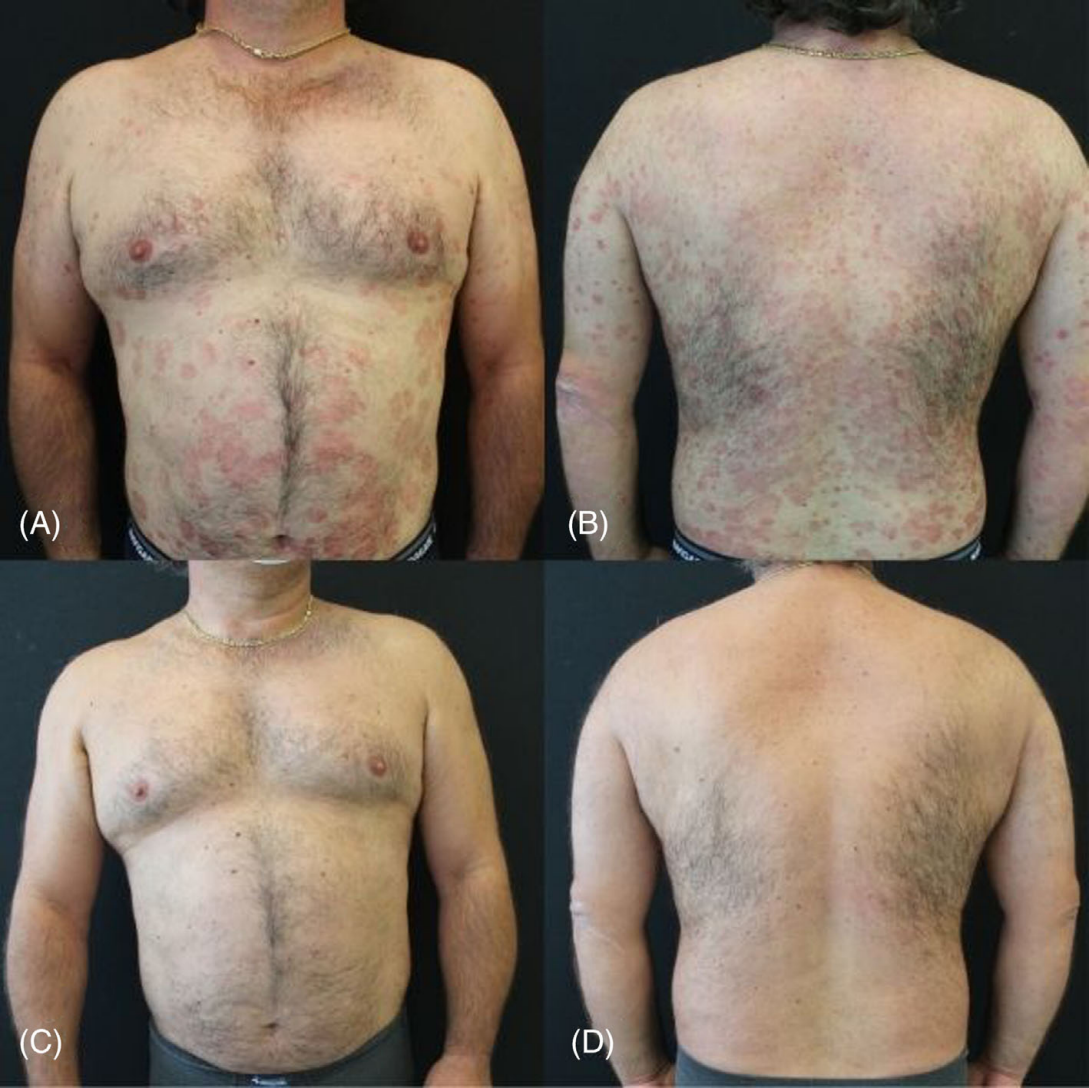
Clinical improvement of a tildrakizumab‐treated patient from baseline (A and B) to week 28 (C and D)

In our population, mean PASI progressively decreased until week 28, with values of 8.11 ± 5.9 at week 4, 3.49 ± 3.65 at week 16 and 1.76 ± 2.82 at week 28, with an overall mean PASI percentage reduction of 88% from baseline to week 28 (Figures 1 and 2), with a *p*‐value <0.0001 for each considered time‐point. When considering absolute PASI, 6 out of 69 patients (10.20%) at week 4, 30 out of 59 patients (50.80%) at week 16 and 47 out of 59 patients (79.71%) at week 28 had an absolute PASI <3, respectively. Regarding PASI75 and PASI90 response rates, 7 (11.90%) patients achieved a PASI75 response at week 4 and 3 (5.11%) achieved a PASI90. PASI scores continued to improve through to week 16, with 34 of 59 (57.60%) patients achieving PASI75 and 20 (33.91%) patients achieving PASI90. At week 28, 48 (81.40%) patients reached PASI75 and 38 (64.40%) patients reached PASI90.

An analysis showed no substantial associations between the considered variables and the efficacy of tildrakizumab at each evaluated time‐point. In particular, the odds of obtain a PASI75 or a PASI90 response were not influenced by gender, BMI >30, PASI >15 at baseline and a prior exposition to biological treatment (all *p*‐values >0.05). No significant differences were recorded even when analyzing the average percentage of improvement of PASI score among the various categories of patients or when PASI and BMI were analyzed as continues variables (data not shown). Data regarding the univariate analysis on PASI75 and PASI90 responses at week 28 according to gender, baseline PASI, BMI and previous biologic experience are reported in Table S1. Multivariate analysis was not performed due to the small number of patients included.

Treatment suspension was recorded in 5 (8.47%) patients; 3 patients experienced primary inefficacy and suspended the treatment at week 28, while 2 patients stopped treatment after the 28‐weeks observation period. One patient who discontinued due to primary inefficacy also experienced the onset of PsA at week 28. No other mild or serious safety issues or discontinuations related to AEs were reported.

## 4 DISCUSSION

This multicenter, retrospective study analyzed a cohort of patients from Lazio region affected by moderate to severe psoriasis and treated with tildrakizumab at standard dosage, with a follow‐up period of 28 weeks. Our findings confirm the efficacy and safety of tildrakizumab in psoriatic patients in line with the findings of RCTs, showing a substantial reduction of PASI scores at the considered time‐points (mean PASI score decreasing from 14.19 ± 8.11 at baseline to 1.76 ± 2.82 at week 28) without reported safety issues. In our study population, PASI75 and PASI90 responses rates at week 28 (81.0% and 64.4%, respectively) are comparable to those reported in RCTs,^3^ in which 80.4% of the patients in the reSURFACE 1 trial and 73.5% in the reSURFACE 2 trial reached a PASI75 response after 7 months of therapy. The proportion of patients achieving a PASI90 response at week 28 was found slightly higher in our study respect to data reported in reSURFACE‐1 and ‐2 trials (64.4% vs. 51.6% and 55.6%, respectively), but the small sample size of our study compared to those of RCTs can be a possible explanation of this difference.

Similar findings are presented by other real‐life experiences with tildrakizumab, although few studies concerning the efficacy and safety of this drug in a real‐world setting are currently available, as previously stated. Burlando et al.^4^ results highlighted the efficacy and safety of tildrakizumab in a retrospective study in a cohort of 26 psoriatic patients followed up to 24 weeks, showing a significant decrease in mean PASI score from baseline (12.5 ± 6.5) to week 24 (0.6 ± 2.1), with a PASI90 response achieved from 91% of patients at week 24. These efficacy rates are higher when compared to those reported from RCTs and those derived from our analysis, but the small sample size and the high proportion (80.8%) of naïve patients could provide an explanation. In a recent Spanish real‐life study, Galán‐Gutierrez et al.^5^ analyzed data from 24 psoriatic patients treated with tildrakizumab with a follow up period of 24 weeks, reporting efficacy rates comparable to those retrieved from reSURFACE 1 and 2 despite using only PASI75 as efficacy endpoint. Of note, this study also highlighted a positive impact on pruritus and an overall improvement of quality of life. Lastly, Ruggiero et al.^6^ recently published a real‐life experience regarding the use of IL23‐inhibitors (guselkumab, risankizumab and tildrakizumab) in psoriatic patients with a follow up period up to 40–44 weeks; tildrakizumab‐treated patients represented a small proportion of the study population (6 out of 34 patients, 17.6%) and the follow up period was limited to 28 weeks, but tildrakizumab still demonstrated good efficacy rates and a favorable safety profile.

To our knowledge, few data are currently available regarding the potential influence of patient‐ or disease‐related variables on the efficacy of tildrakizumab.

A recent review analyzing data from phase 2b/3 trials (P05495, reSURFACE 1 and 2)^8^ stratified patients in subgroups defined by age, gender, race, weight, psoriatic arthritis and prior traditional systemic treatment or biologic use, with no significant differences in terms of efficacy among these categories of tildrakizumab‐treated patients. Indeed, our results showed that the clinical response to tildrakizumab does not appear to be influenced by the demographic and clinical characteristics of the treated patients even in a real‐life setting, allowing continuous improvement of skin lesions over time. In our study, BMI did not affect the efficacy of tildrakizumab, which was found to be similar in both patients with BMI <30 and patients with BMI ≥30, consistent with the results of Burlando et al and Galán‐Gutierrez et al.^4^, ^5^ In partial contrast with our findings and with those reported in the above‐mentioned real‐life study, pooled data retrieved from phase 2 and phase 3 tildrakizumab trials^9^ showed that PASI and Physician Global Assessment (PGA) responses to tildrakizumab were greater in patients with lower body weight versus higher body weight at week 12. However, the short time‐period considered could represent a limitation, not taking into account potential long‐term improvement of clinical response. PASI at baseline also did not impact the efficacy of tildrakizumab in our study population similarly to the results found in reSURFACE 1 and 2.

Finally, our study covered an observation period of up to 28 weeks. Over this time frame, although not sufficient for an accurate analysis of long‐term efficacy and safety, tildrakizumab was shown to confer a sustained response over time along with a favorable safety profile. These results are in line with what was reported by the analysis up to 148 weeks of reSURFACE 1 and 2 trials,^10^ where tildrakizumab showed significant long‐term maintained response rates without safety issues. A possible explanation could lie in the impact of IL‐23 inhibition on immune memory, as this interleukin's action seems to promote the pathogenic activity of tissue‐resident memory cells implicated in both the inflammatory cascade of psoriasis and its potential relapses.^11^, ^12^ Because of the short period of observation, no definitive conclusions regarding long‐term efficacy of tildrakizumab can be made, and further studies with longer observation periods are needed to confirm this finding.

### 4.1 Limitations

The limitations of this study are mostly represented by the retrospective method of data collection and the absence of a control group, together with the relatively small sample size and the observation period limited to 28 weeks.

### 4.2 Conclusions

In conclusion, our real‐life experience with tildrakizumab confirms its efficacy and safety regardless of external variables such as gender, BMI, baseline disease severity or previous use of biological therapies, confirming data from RCTs and highlighting the potential use of tildrakizumab with high‐efficacy rates in patients with unfavorable characteristics.

## CONFLICT OF INTEREST

The authors declare no conflict of interest.

## Supporting information


**TABLE S1**: Analysis regarding influence of patient‐ or disease‐related variables on PASI75 and PASI90 responses.

## Data Availability

The data that support the findings of this study are available from the corresponding author upon reasonable request.
