# Supplementary material for: Tildrakizumab in moderate‐to‐severe plaque psoriasis: A multicenter, retrospective, real‐life study
Source: Dermatol Ther. 2022 Apr 11;35(6):e15488. doi: 10.1111/dth.15488 (PMC9287013; doi:10.1111/dth.15488)
Supplement: Supplementary file 1 — TABLE S1: Analysis regarding influence of patient‐ or disease‐related variables on PASI75 and PASI90 responses. [file DTH-35-0-s001.docx]

**SUPPLEMENTARY TABLES (DTH-21-2337)**

**Supplementary Table 1 –analysis regarding influence of patient- or disease-related variables on PASI75 and PASI90 responses**

|  | | **Week 28** | | | |
| --- | --- | --- | --- | --- | --- |
|  |  | **PASI75** | ***P*** | **PASI90** | ***P*** |
| **All patients** | | 48 (81.4) | n/a | 38 (64.4) | n/a |
| **Gender** | **Male** | 28 (82.4) | 0.820 | 22 (64.7) | 0.956 |
|  | **Female** | 20 (80.0) |  | 16 (64.0) |  |
| **BMI** | **<30** | 32 (84.2) | 0.453 | 24 (63.2) | 0.789 |
|  | **≥30** | 16 (76.2) |  | 14 (66.7) |  |
| **Baseline PASI** | **<15** | 32 (78.0) | 0.329 | 25 (61.0) | 0.410 |
|  | **≥15** | 16 (88.9) |  | 13 (72.2) |  |
| **Previous therapies** | **Bio-naïve** | 29 (85.3) | 0.369 | 23 (67.6) | 0.548 |
|  | **Bio-experienced** | 19 (76.0) |  | 15 (60.0) |  |

PASI: Psoriasis Area and Severity Index; BMI: Body Mass Index; n/a: not applicable
